# Syneresis in Gels of Highly Ferulated Arabinoxylans: Characterization of Covalent Cross-Linking, Rheology, and Microstructure

**DOI:** 10.3390/polym9050164

**Published:** 2017-05-05

**Authors:** Ana M. Morales-Burgos, Elizabeth Carvajal-Millan, Yolanda L. López-Franco, Agustín Rascón-Chu, Jaime Lizardi-Mendoza, Norberto Sotelo-Cruz, Francisco Brown-Bojórquez, Alexel Burgara-Estrella, Martin Pedroza-Montero

**Affiliations:** 1 Research Center for Food and Development, CIAD, A.C. Carretera a La Victoria Km. 0.6, Hermosillo, Sonora 83304, Mexico; ana.morales@estudiantes.ciad.mx (A.M.M.-B.); lopezf@ciad.mx (Y.L.L.-F.); arascon@ciad.mx (A.R.-C.); jalim@ciad.mx (J.L.-M.); 2 Department of Medicine, University of Sonora, Rosales y Blvd. Luis D. Colosio, Hermosillo, Sonora 83000, Mexico; norberto.sotelo@unison.mx; 3 Department of Polymers and Materials, University of Sonora, Rosales y Blvd. Luis D. Colosio, Hermosillo, Sonora 83000, Mexico; fbrown@guaymas.uson.mx; 4 Department of Physics Research, University of Sonora, Rosales y Blvd. Luis D. Colosio, Hermosillo, Sonora 83000, Mexico; alexel.burgara@unison.mx (A.B.-E.); mpedroza@cifus.uson.mx (M.P.-M.)

**Keywords:** arabinoxylan gel, ferulic acid, syneresis, maize bran

## Abstract

Arabinoxylans (AXs) with high ferulic acid (FA) content (7.18 µg/mg AXs) were cross-linked using laccase. Storage (*G*’) modulus of AX solutions at 1% (AX-1) and 2% (AX-2) (*w*/*v*) registered maximum values of 409 Pa and 889 Pa at 180 min and 83 min, respectively. Atomic force microscopy revealed the grained and irregular surface of the AX-1 gel and the smoother surface without significant depressions of the AX-2 gel. Cured AX gels exhibited a liquid phase surrounding the samples indicating syneresis. The syneresis ratio percentage (% *R*_s_) of the gels was registered over time reaching stabilization at 20 h. The % *R*_s_ was not significantly different between AX-1 (60.0%) and AX-2 (62.8%) gels. After 20 h of syneresis development, the dimers of the FA in the AX-1 and AX-2 gels significantly increased by 9% and 78%, respectively; moreover, the trimers of the FA in the AX-1 and AX-2 gels, by 94% and 300%, respectively. Scanning electron microscopy showed that, after syneresis stabilization, AX gels presented a more compact microstructure. Syneresis development in the gels of highly ferulated AXs could be related to the polymer network contraction due to the additional formation of dimers and trimers of the FA (cross-linking structures), which may act like a “zipping” process, increasing the polymer chains′ connectivity.

## 1. Introduction

**Figure 1:**
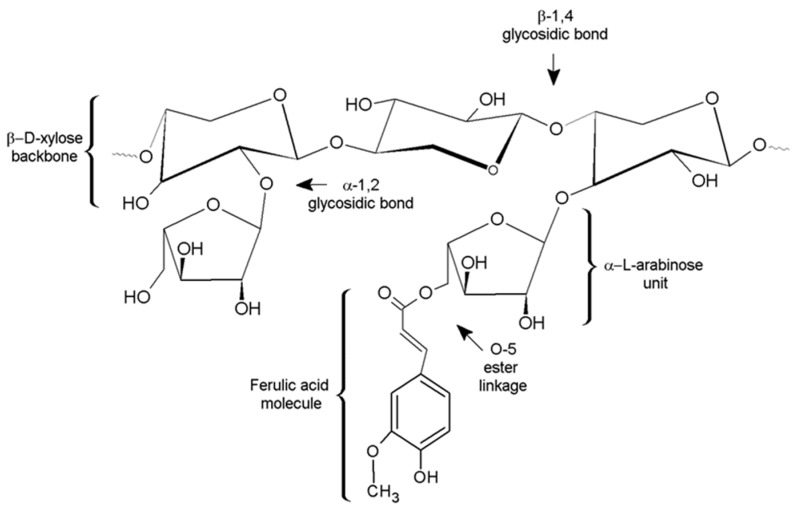
The chemical structure of a representative fraction of ferulated arabinoxylans (AXs).

Arabinoxylans (AXs) are hemicellulose polysaccharides constituted by a linear backbone of β-(1–4)-linked d-xylopyranosyl units to which α-l-arabinofuranosyl units are attached trough α-(1–3) and/or α-(1–2) linkages (Figure 1). In addition, ferulic acid (FA) (3-methoxy, 4 hydroxy cinnamic acid) molecules may be ester-linked on the (O)-5 position to some of the arabinose residues. Some other components like galactose, glucoronic acid, and *p*-coumaric acid may exist in AX side chains [1].

AXs show interesting functional properties as thickeners, stabilizers, and emulsifiers [2], they form highly viscous solutions, and one of the most important properties is their ability to form chemical gels through covalent cross-linking of FA upon oxidation by some chemical (ferric chloride or ammonium persulfate) or enzymatic (peroxidase/H_2_O_2_ or laccase/O_2_) agents [2]. In addition to covalent crosslinks, some physical interactions between AX chains, e.g., hydrogen bonds, may be involved in gel formation [2]. The oxidation of FA allows the coupling of AX chains through the formation of dimers and trimers of FA (di-FA and tri-FA, respectively). In AX gels, coupling gives rise to five isomeric forms of di-FA (5-5′–, 8-5′-benzo–, 8-*O*-4′–, 8-5′–, and 8-8′–) and one tri-FA structure (4-*O*-8′, 5-5′–) [2]. Regarding the FA content in AX, it has been reported to be in the range from 0.01 up to 2.3 μg/mg AXs [3,4]. Previous reports by Carvajal-Millan et al. [5,6] have demonstrated that increasing FA content in AXs results in more covalent crosslinks and a more compact structure in the gels. Covalent cross-linked AX gels have been described as gels that “exhibit no syneresis after a long period of storage” [7,8], but to our knowledge the effect of high FA content on AXs (content above those previously reported in the literature) has not yet been studied and may modify the gel characteristics as many covalent crosslinks will be formed. Syneresis is an instability phenomenon described as a “spontaneous contraction of a gel accompanied by the expulsion of liquid from the pores” [9]. One of the most common examples is the cheese curd, but this phenomenon has been reported in some protein gels including gelatin [10] and casein gels [11], as well as in polysaccharide gels such as agar [12,13], carrageenan [14,15], and chitin [16], among others. The aim of the present work was to extract, characterize, and cross-link AXs with high FA content (above those reported in the literature) and to study the syneresis phenomenon in the gels formed. 

## 2. Materials and Methods

### 2.1 Materials

AXs were extracted from maize bran kindly provided by an agricultural company located in Sinaloa, Mexico. The maize bran provided was isolated from the kernel by a mechanical process, and no chemical or enzymatic treatment was used to separate the bran. Commercial laccase (E.C. 1.10.3.2) from *Trametes versicolor*, α-amylase (E.C. 3.2.1.1), amyloglucosidase (E.C. 3.2.1.3), and pronase E and other chemical reagents were purchased from Sigma Chemical Co. (St. Louis, MO, USA).

### 2.2 Methods

#### 2.2.1. Extraction of AXs

AXs were extracted following enzymatic and alkali procedures described by Carvajal-Millan et al. [5,17].

#### 2.2.2. Neutral Sugars

Neutral sugar content in purified AXs was determined by gas chromatography (6890N GC, Agilent Technologies, Santa Clara, CA, USA) with a flame ionization detector and a DB 225 capillary column (30 m × 0.32 mm internal diameter, 0.15 μm film thickness; Agilent Technologies, Santa Clara, CA, USA) after hydrolysis and derivatization into alditol acetates as described elsewhere [18,19]. 

#### 2.2.3. Ferulic Acid (FA), Dimers of Ferulic Acid (di-FA), and Trimers of Ferulic Acid (tri-FA) in AXs and AX Gels

Concentrations of FA, di-FA, and tri-FA in AXs and AX-1 and AX-2 gels were determined by RP-HPLC using an Alltima C18 column (250 mm × 4.6 mm; Alltech Associates, Inc., Deerfield, IL, USA) and a photodiode array detector (Waters 996; Waters Corporation, Milford, MA, USA) after a de-esterification step as described elsewhere [2,18].

#### 2.2.4. Rheological Measurements

AX gel formation was rheologically investigated by a small amplitude oscillatory shear. Cold (4 °C) AX solutions (1% and 2% *w*/*v*) in a 0.1 M sodium acetate buffer (pH 5.5) were mixed with laccase (1.675 nkat per mg AX) and immediately poured onto parallel-plate geometry (40 mm in diameter) of a strain-controlled rheometer (Discovery HR-3 rheometer; TA Instruments, New Castle, DE, USA) maintained at 4 °C. Exposed edges were recovered with silicone to prevent evaporation. AX gelation was started by a rapid increase in temperature from 4 to 25 °C and monitored at 25 °C by following the storage (*G*′) and loss (*G*′′) modulus. All measurements were carried out at a frequency of 0.25 Hz and 5% strain (linearity range of viscoelastic behavior) during 180 min. Frequency sweep (0.01 to 10 Hz) at 5% strain and 25 °C was conducted out at the end of the network formation.

#### 2.2.5. Atomic Force Microscopy

Atomic force microscopy (AFM) was used to characterize the topographical features of AX-1 and AX-2 gels. Films were prepared by dissolving AXs in a 0.1 M sodium acetate buffer (pH 5.5), and laccase (1.675 nkat per mg AX) was then added. One drop of each solution was immediately deposited on freshly cleaved mica and allowed to develop in Petri dishes for 1 h at 25 °C under 100% relative humidity. After gelation, the samples were allowed to dry in air. AX-1 and AX-2 film images were obtained by using an XE-Bio atomic force microscope (Park Systems, Suwon, Korea) in non-contact mode under atmospheric conditions and using a PPP-NCHR cantilever. The images were processed using XEI-AFM software (Park Systems, Suwon, Korea). Root mean square roughness (*R*_q_) and heights values were measured over 5 µm × 5 µm areas of films.

#### 2.2.6. Syneresis

A mixture of 2 mL of AX-1 and AX-2 solutions prepared in a 0.1 M sodium acetate buffer (pH 5.5)/laccase (1.675 nkat per mg AX) was rapidly poured to a 5 mL tip-cutoff syringe (diameter 1.5 cm) and allowed to gel for 60 min at 25 °C. After gelation, the gels remained in the syringe where syneresis development was monitored at 25 °C, as described by Ako [15]. The once removal method (ORM) syneresis measurement was performed on the sample: the solvent released was periodically taken out and the gel weigh was recorded; the removed solvent was then taken back to the gel and allowed to remain in contact until the next measurement. After 20 h, the gel samples were weighed, and the syneresis ratio percentage (% *R*_s_) was calculated using the following equation:

$$\%\;R_{s}=\frac{W_{e}}{W_{g}}\times 100$$

where $W_{e}$ is the weight of solvent released by the gels, and $W_{g}$ is the initial weight of AX gel. All measurements were made at room temperature. 

#### 2.2.7. Swelling

At the end of the syneresis experiment, the AX-1 and AX-2 gels were removed from the syringes, placed in glass vials, and weighed. Subsequently, they were allowed to swell at 25 °C as described by Carvajal-Millan et al. [5]. After 12 h, the samples were taken out, blotted, and weighed. The equilibrium swelling was reached when the weight of the samples changed by no more than 3%. The swelling ratio (*q*) was calculated using the following equation:

$$q=\frac{W_{S}-W_{\mathrm{AX}}}{W_{\mathrm{AX}}}$$

where $W_{S}$ is the weight of swollen gels, and $W_{\mathrm{AX}}$ is the weight of the AXs in AX-1 or AX-2 gels. 

#### 2.2.8. Scanning Electron Microscopy

AX-1 and AX-2 gels were frozen by liquid nitrogen immersion and then lyophilized at −37 °C/0.133 mbar in a Freezone 6 freeze drier (Labconco, Kansas, MO, USA). Lyophilized gels were coated with a thin layer of gold, and the microstructures were studied by scanning electron microscopy (SEM) (JEOL 5410LV, JEOL, Peabody, MA, USA) at low voltage (20 kV). SEM images were obtained in secondary electron image modes (SEI).

#### 2.2.9. Statistical Analysis

Small deformation measurements were made in duplicates, while neutral sugars, phenolic acids, syneresis, and swelling experiments were performed in triplicates. All results are expressed as mean ± SD.

## 3. Results and Discussion

### 3.1. Extraction and Characterization of AXs

AXs were extracted from 5.5 kg of maize bran. The AX yield was 1.04% (*w*/*w*) on a dry matter basis (db, *w* AX/*w* maize bran). This yield is low compared to the values reported in the literature for maize bran [17,20], possibly due to the short period of alkali extraction used in the present work (30 min). It has been proposed that an increase in alkaline extraction time produces a higher AX yield but negatively affects the FA content and consequently their gelling capability [5,17]. The composition of the AXs is presented in Table 1. 

**Figure 2:**
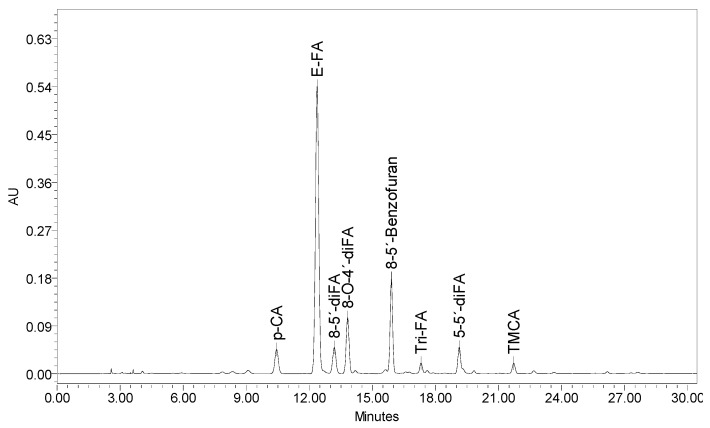
HPLC chromatogram of phenolic acids profile for a representative AX sample monitored at 320 nm.

The total carbohydrate content was 96% with an AX purity of 82%, obtained from the sum of xylose + arabinose on a dry basis. The arabinose-to-xylose ratio (A/X) was 0.72, which is similar to other AXs extracted from maize bran (0.75–0.85) [17,20]. The A/X obtained in the present study is indicative of moderately branched AXs. Galactose, glucose, and mannose were also found in AXs. The galactose content may arise from AX side-chains, while glucose is likely a remnant originated by a de-starch step used in extraction procedures [21]. An important feature found in AXs was a high FA content (7.18 ± 0.20 μg/mg AX), which is considerably higher than others reported for maize bran AXs [17,20]. AXs showed the presence of di-FA (0.44 ± 0.02 μg/mg AX) and tri-FA (0.01 ± 0.002 μg/mg AX), suggesting that AX chains might be intra- and/or inter- cross-linked. The relative percentages of each one of the different di-FA present in AXs were as follows: 69, 16, and 15% for the 8-5′ (mainly in benzofuran form), 8-*O*-4′, and 5-5′ structures, respectively. The predominance of 8-5′-benzo di-FA has been previously reported in AXs from maize bran [20] and wheat endosperm [5,18]. A representative HPLC chromatogram for phenolic acid analysis is given in Figure 2.

### 3.2. Rheology

**Figure 3:**
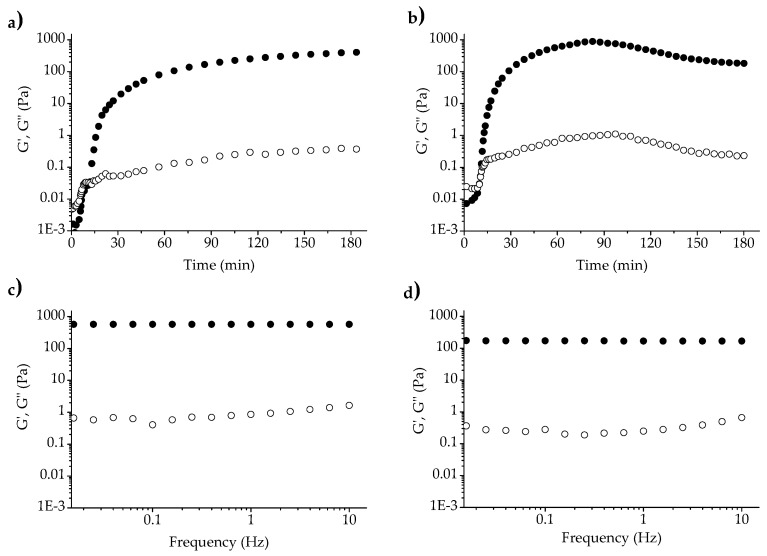
Kinetics of gelation (**a**,**b**) and mechanical spectra (**c**,**d**) of AX-1 gel and AX-2 gel, respectively. *G*′ (●), *G*′′ (◯). Measurements at 25 °C.

AX solutions at 1% (AX-1) and 2% (*w*/*v*) (AX-2) exhibited oxidative gelation under laccase exposure. The kinetics of the gelation processes were rheologically monitored by a small amplitude oscillatory shear following the storage (*G*′) and loss (*G*′′) moduli over time. Figure 3a,b show the gelation profile for AX-1 and AX-2; at the beginning, *G*′′ was higher than *G*′, but the increase in *G*′ was faster and prevailed over *G*′′. Gelation times of 16 min and 10 min for AX-1 and AX-2, respectively, were calculated from the crossover of *G*′ and *G*′′ curves, which indicate the sol/gel transition point. The AX-1 and AX-2 gels attained maximum *G*′ values of 409 and 889 Pa at 180 and 83 min, correspondingly. In previous studies [2,3,22], lower *G*′ values have been reported for maize AX gel (2–328 Pa) at higher polysaccharide concentrations (4%–10% *w*/*v*); the high *G*′ values registered in the present work using low AX concentrations (1% and 2% *w*/*v*) could be attributed to the higher FA content in AXs (7.18 μg/mg AXs) in comparison to the maize AXs used in those previous studies (0.01–0.34 μg/mg AXs). In the literature, rheological monitoring of AX gels has been reported to reach a plateau region for *G*’ after 60 or 300 min of cross-linking reactions [18,22]. However, in the present study, an unexpected behavior was observed in AX-2 as a “steady-state” of gel was not achieved; *G*′ reached a maximum value (889 Pa at 83 min) and it then started to decrease, presenting a value of 182 Pa at 180 min. This G′ reduction in AX-2 gel was attributed to the expulsed liquid observed around the sample once the parallel-plate geometry was removed, likely reflecting the syneresis event, rather than the loss of network structure or damage.

The mechanical spectra of AX-1 and AX-2 gels are shown in Figure 3c,d, respectively. The *G*′ value was higher and prevailed over *G*′′ for both samples, *G*′ was also independent of frequency range, while *G*′′ was slightly dependent. These mechanical spectra are typical of a solid-like material and have been previously described for AX gels [5,22].

### 3.3. Atomic Force Microscopy (AFM)

**Figure 4:**
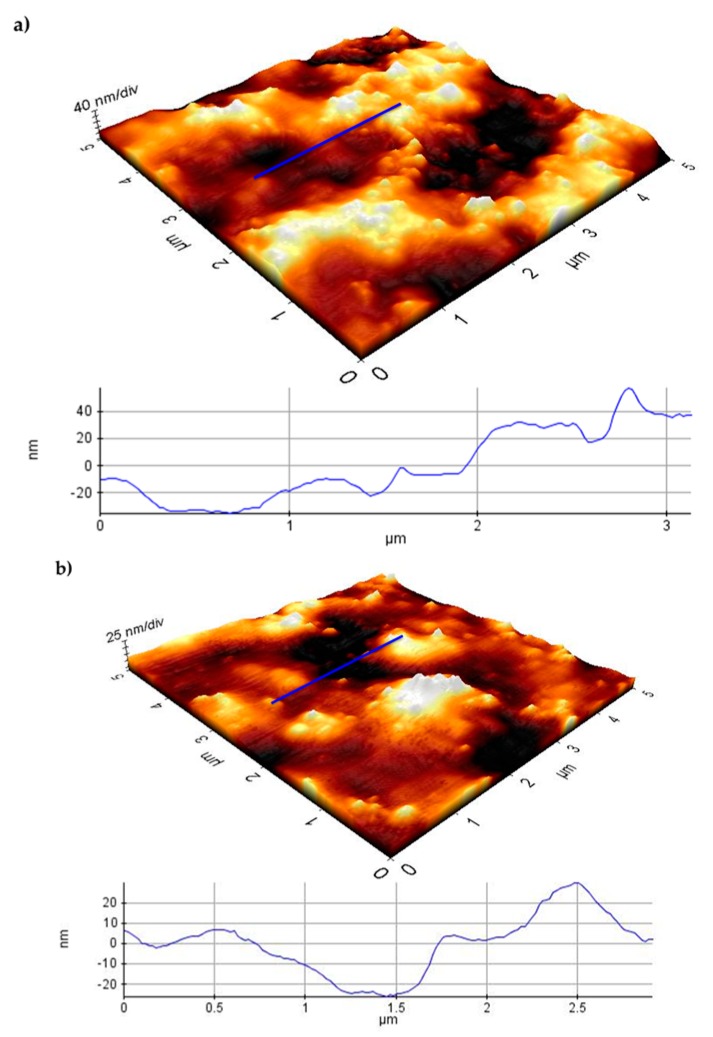

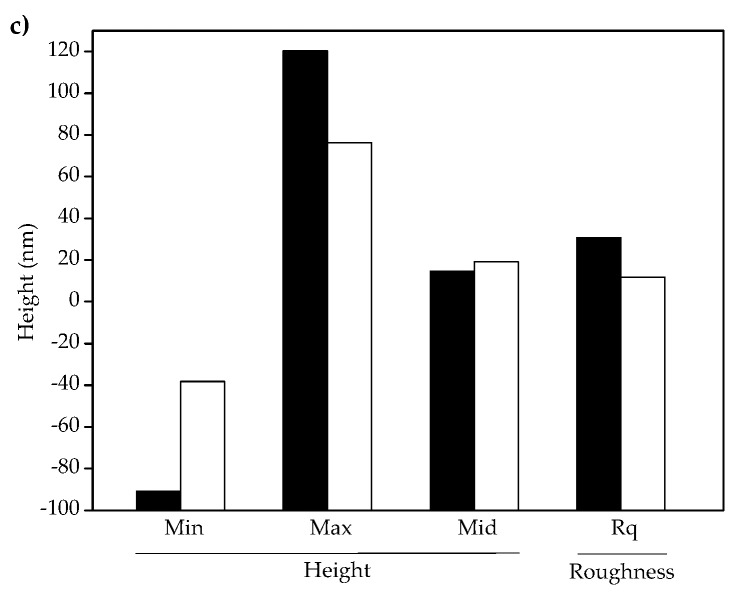
AFM images showing the surface and profile line of AX-1 (**a**) and AX-2 (**b**) gels (5 µm × 5 µm). Graphical representation of height values for AX-1 (black bars) and AX-2 (white bars) films (**c**).

The topography of AX-1 and AX-2 dried gels was investigated by AFM, and the results are depicted in Figure 4a,b, respectively. This analysis has revealed that the surface morphology slightly changed when the gel was formed at 1% or 2% (*w*/*v*) in AXs. It was observed that AX-1 gel showed a grained and irregular surface, while AX-2 gel presented a less grained and more regular surface without larger hollows or embossments as described before by Velkova et al. [23] for AX films. Root mean square roughness (*R*_q_) values were 30.702 and 11.761 nm for the total surface of the AX-1 and AX-2 films, respectively, which confirms previous observations. Height characteristics extracted from the images are presented in Figure 4c.

### 3.4. Syneresis

**Figure 5:**
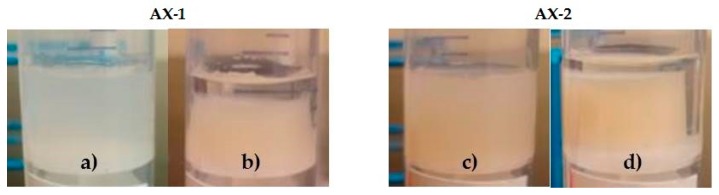
AX-1 and AX-2 gels before ((**a**,**c**), respectively) and after ((**b**,**d**), respectively) 20 h showing syneresis development.

Macroscopic observation of both gels (AX-1 and AX-2) after 20 h of syneresis development clearly showed a liquid phase surrounding the samples (Figure 5). 

**Figure 6:**
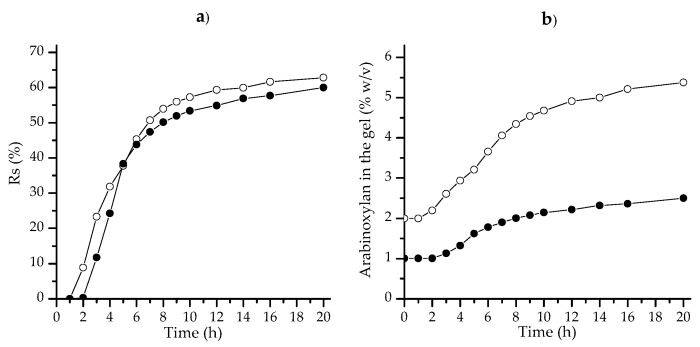
(**a**) Syneresis of AX-1 (●) and AX-2 (◯) gels as a function of time. (**b**) Changes in AX concentration in the gels versus time occurring during syneresis.

The syneresis ratio percentage (% *R*_s_) of gels describes the amount of solvent released from the gel network. The Rs percentage of AX-1 and AX-2 gels after 1 h of laccase exposure (near the plateau region in Figure 3a,b) was registered for 20 h at 25 °C. Changes in % *R*_s_ and AX concentration in the gels were calculated from the once removal method (ORM), and the results are presented in Figure 6. Ako [15] has reported that the ORM may allow the gel to stabilize in its fluid; in the present study, % *R*_s_ stabilization occurred after 20 h. Some authors [15,16] have reported that increasing the polymer concentration decreases the syneresis ratio as a result of the corresponding increase in elasticity. Nevertheless, in the present work, % *R*_s_ values at the end of the test were 60.0 ± 0.9 and 62.8 ± 0.6 for the AX-1 and AX-2 gels, respectively, and thus were not significantly different.

### 3.5. Swelling

**Figure 7:**
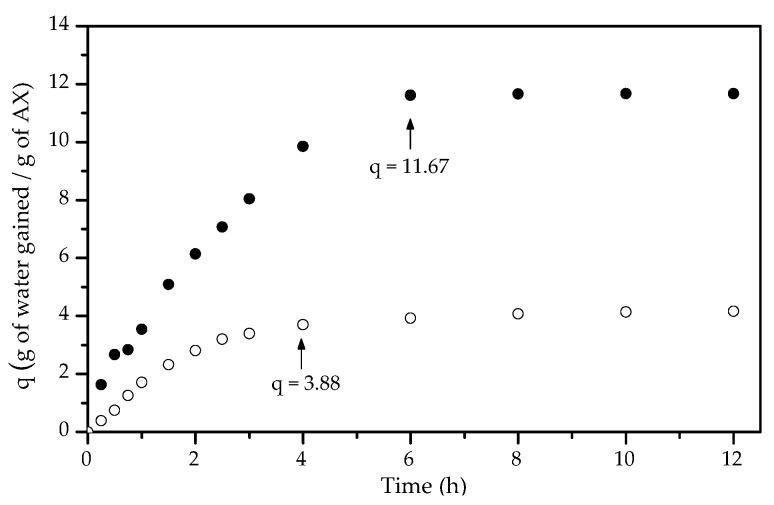
Swelling of AX-1 (●) and AX-2 (◯) gels after % *R*_s_ stabilization. Tests in sodium azide (0.02% *w*/*v*) at 25 °C. Arrows indicate the mean *q* value at the equilibrium swelling.

The swelling ratio (*q*) of gels describes the amount of water stored within the gel network. The equilibrium swelling of AX-1 and AX-2 gels was analyzed in gels after % *R*_s_ stabilization (20 h after laccase-induced gelation for both gels). Swelling experiments before % *R*_s_ stabilization could not be determined if changes in the weight of the gel were due to liquid entrance (swelling), expulsion (syneresis), or both. The equilibrium swelling time and *q* value were 6 h and 4 h and 11.67 ± 0.15 and 3.88 ± 0.08 g water gained/g AXs for AX-1 and AX-2 gels, respectively (Figure 7). This difference could be related to the weaker structure of the AX-1 network as a result of the lower polysaccharide concentration, while AX-2 gel presented a more compact structure that restricts water uptake; a similar behavior has been previously reported by Carvajal-Millan et al. [6] for laccase-induced wheat AX gels at different polysaccharide concentrations.

### 3.6. Covalent Cross-Links (di-FA and tri-FA)

Table 2 shows the FA, di-FA, and tri-FA content as well as the percentage of FA oxidized and FA recovered in the AX-1 and AX-2 gels before syneresis started and after % *R*_s_ stabilization (20 h). The FA content in the gels decreased in 47% and 62% for AX-1 and AX-2 gels, respectively after % *R*_s_ stabilization. A similar value (59%) was found for wheat AX gels at 1% (*w*/*v*) after 26 h of aging, but syneresis development was not reported in those gels, probably due to the lower FA content in the AX sample (1.7 µg/mg AX) used [7].

di-FA and tri-FA contents in AX-1 and AX-2 gels ranged between 1.24 and 2.71 µg/mg AXs and from 0.07 to 0.31 µg/mg AXs, respectively, which are higher than those reported in the literature (di-FA 0.030 µg/mg AXs, tri-FA 0.015 µg/mg AXs) for maize AX gels at the same polysaccharide concentrations (1% and 2% *w*/*v*) but lower (0.34 µg/mg AX) FA content in the molecule [17]. In the present study, an increase in AX concentration in the gel from 1% to 2% (*w*/*v*) resulted in significantly enhanced concentrations of di-FA and tri-FA, as previously reported for other AX gels in the literature [6]. 

After % *R*_s_ stabilization, the di-FA for AX-1 and AX-2 gels increased by 9% and 78%, respectively; moreover, the tri-FA in AX-1 and AX-2 gels increased by 94% and 300%, respectively. The percentage of FA oxidized in the gels increased after % *R*_s_ stabilization, as both FA and O_2_ were still accessible to the enzyme in the gel and laccase is able to diffuse through the AX gel network [7]. This additional FA oxidation could explain the increase in di-FA and tri-FA content after 20 h of laccase exposure. It is possible that, after initial gelation, there is a self-amplifying effect with FA units from different AX chains forming di-FA and tri-FA, bringing polymer chains closer together and triggering more FA oxidation. Such a mechanism could form additional cross-linking structures, acting like a “zipping” process increasing the polymer chains connectivity and the polymer network contraction, resulting in the syneresis phenomenon.

In gels formed with AXs displaying a lower FA content (1.7 µg/mg AXs), a decrease in di-FA (42%) and tri-FA (34%) content was registered after 26 h of aging in spite of FA oxidation [7]. Carvajal-Millan et al. [7] proposed a free radical mechanism initiated by laccase where the phenoxy radicals produced underwent secondary reactions resulting in a loss of AX crosslinking bonds. It is possible that, at a longer incubation time (>20 h after gelation), the AX-1 and AX-2 samples would also exhibit a decrease in cross-links structures, but this was not investigated in the present study. 

It is important to note that, for all samples analyzed in the present study, the FA recovered (calculated by the sum of di-FA and tri-FA content in gels with respect to the oxidized FA) was never equal to the extent of the FA oxidized, and this result has been previously reported in other AX gels [7,18]. The authors associated with those studies suggest that one part of the FA that is oxidized after AX gelation is probably transformed into ferulate cross-linking structures other than di-FA and tri-FA. For all AX gels of the present study, the main di-FA structure was 8-5′ (mainly benzofuran form) (~70%) followed by 8-*O*-4 (~20%) and 5-5′ (~10%). The di-FA 8-5′ benzofuran form was also the structure that presented a higher increase in relation to the di-FA content in AXs before gelation. Similar results showing 8-5′ di-FA as the main di-FA in AX gels have been previously reported [7]. The percentages of oxidized FA were augmented when the time after laccase exposure increased, reaching maximum values (69%–74%) at 20 h. 

### 3.7. Scanning Electron Microscopy (SEM)

**Figure 8:**
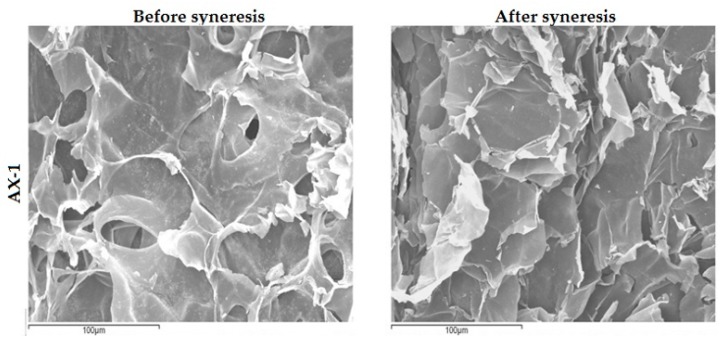

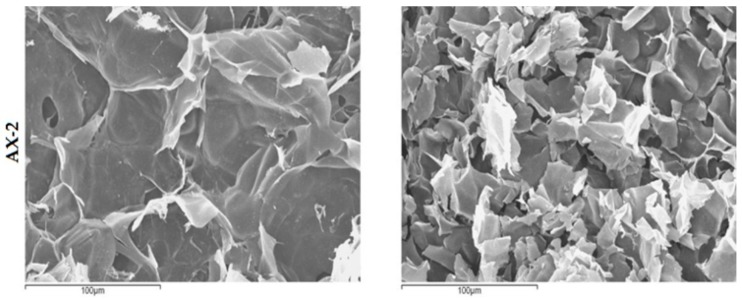
SEM images showing AX-1 and AX-2 gels microstructure at 500× amplification before (**left**) and after (**right**) syneresis phenomenon.

SEM images of gels at 500× magnification are shown in Figure 8. Before syneresis, AX-1 presented an irregular and highly porous microstructure, which might be due to the low concentration used for gel formation. After syneresis (20 h after laccase exposure), the AX-1 gel presented a more compact but still heterogeneous microstructure. The microstructure of the AX-1 gel after syneresis was similar to the microstructure observed in the AX-2 gel before syneresis, and both resemble an irregular honeycomb structure with diffusion pathways that are not straightforward. This similarity could be attributed to the increase in concentration and covalent cross-linking structures in the AX-1 gel after syneresis, as observed in Figure 6b and Table 2. The AX-2 gel after syneresis featured many connections and a more compact microstructure when compared to the AX-2 gel before syneresis, which is also consistent with the syneresis phenomenon. These microstructures are similar to those reported previously by Paës and Chabbert [24].

## 4. Conclusions

Arabinoxylans (AXs) with high ferulic acid concentrations form covalent gels after laccase induction and display high G′ values. Nevertheless, these covalent gels undergo syneresis during gel aging. Covalent cross-link (dimers and trimers of ferulic acid) content in these AX gels increased during syneresis development. It is possible that, after initial gelation, AX chains crosslinked through di-FA or tri-FA are brought closer together, and the oxidation of ferulic acid remaining in the polysaccharide chains act like a “zipping” that increases the polymer network connectivity, resulting in syneresis phenomena. In the present study, AX gels at 1% and 2% (*w*/*v*) did not exhibit differences in the syneresis ratio, but they varied in the swelling ratio (*q*), the topography, and the microstructure, with the higher polysaccharide concentration resulting in a lower *q* value, a less grained surface, and a more compact and regular microstructure. To our knowledge, this is the first report on syneresis for arabinoxylan gels. 

## Figures and Tables

**Table 1:** Composition of maize bran AXs.

Component	Content
Arabinose ^1^	34.50 ± 2.94
Xylose ^1^	47.79 ± 4.50
Galactose ^1^	8.18 ± 0.71
Glucose ^1^	5.26 ± 0.56
Mannose ^1^	0.53 ± 0.03
Ferulic acid ^2^	7.18 ± 0.20
di-FA ^2^	0.44 ± 0.02
tri-FA ^2^	0.01 ± 0.002

^1^ Results are expressed in g/100 g AXs. ^2^ Phenolic acids are expressed in μg/mg AXs.

**Table 2:** FA, di-FA, and tri-FA content in AX gels before and after syneresis.

Gel	% *R*_s_	FA	di-FA	tri-FA	FA Oxidized (%)	FA Recovered (%)
		(µg/mg AX)				
AX-1	0	4.16 ± 0.08	2.49 ± 0.48	0.16 ± 0.04	42 ± 1	88 ± 2
	60 *	2.21 ± 0.20	2.71 ± 0.79	0.31 ± 0.10	69 ± 3	61 ± 2
AX-2	0	5.00 ± 0.10	1.24 ± 0.28	0.07 ± 0.03	30 ± 1	61 ± 3
	62 *	1.88 ± 0.14	2.21 ± 0.65	0.28 ± 0.05	74 ± 2	47 ± 1

* % *R*_s_ stabilization value at 20 h.
